# On the Resection of the Posterior Spinal Nerve Roots

**Published:** 1911-06

**Authors:** Ernest W. Hey Groves

**Affiliations:** Assistant Surgeon, Bristol General Hospital


The Bristol

Medico-Chirurgical Journal.

*"Scire est nescire, nisi id me
Scire alius sciret."*

JUNE, 1911.

### ON THE RESECTION OF THE POSTERIOR SPINAL NERVE ROOTS.

BY

Ernest W. Hey Groves, M.S., F.R.C.S.,
*Assistant Surgeon, Bristol General Hospital.*

It is now twenty-three years since Abbé in America and Bennett in London first deliberately divided the posterior nerve roots for intractable pain, yet only about forty cases of the operation for this condition have been recorded, and the procedure has not up till very recent times found a regular place in surgical technic. This is due partly no doubt to the rarity of cases suitable for it, but also to the somewhat equivocal results obtained, and to an exaggerated fear of the gravity of the necessary laminectomy. In only about one-third of these cases was the pain definitely stated to have been cured; in many it returned in a modified or similar degree after an interval of some months, and in several, the condition gave place to insanity, hysteria or morphinism. I have had two cases in which I performed the operation for the relief of pain.

**Case 1.**—F. H., a clerk, aged 50. Since 1896 he had suffered from tabes dorsalis, with very severe lightning pains in the left leg, and an extreme degree of Charcot's knee-joint. All sorts of therapeutical remedies had been applied without success. On the 16th August, 1909, I resected the last three lumbar and the first three sacral nerve roots on the left side. On the 30th August, 1909, I excised the disorganised knee-joint *without any anæsthetic whatever*, demonstrating the completeness of the analgesia, for the patient was not conscious of any discomfort throughout. The leg healed perfectly, and he can now use it for walking, which he does in the characteristic tabetic manner. But as regards the pain the operation has only been a partial cure, and he often has attacks of gnawing pain.

**Case 2.**—R. S., aged 39. A married woman with four children. Since 1907 she had severe pain in the left leg following puerperal pyæmia. In 1909 this became so severe that she begged to have the leg amputated. On October 8th, 1909, the posterior roots of the three last lumbar and the first three sacral nerves on the left side were divided. For some few weeks after this she had complete relief from the pain, but after she had returned to her own home the pain returned with renewed intensity, and in April, 1910, she became insane, and had to be removed to the asylum. Within two months both mental condition and complaint of pain became better, and she returned home in October, 1910. Then the same unfortunate sequence of events recurred, first, severe attacks of paroxysmal pain, culminating in insanity in January, 1911, but ever since the operation the leg itself has been completely analgesic.

These cases show very clearly that posterior nerve root division cannot be relied upon for the cure of peripheral pain, which appears to be in many cases of a psychical or central origin.

**Cases of Visceral Crises.**—In 1908 Professor Foerster, of Breslau, suggested that the crises of tabes were due to an exaggerated reflex mechanism, which might be obviated by the division of the posterior roots concerned. In conjunction with Professor Kuttner, he operated with the most brilliant results upon a tabetic aged 47, who for six years had been subject to severe gastric crises of pain and vomiting. Several other cases of a similar nature have been reported from Germany and America, and in all of these the operation cured the vomiting. Unfortunately my own case was only successful from a physiological point of view.

**Case 3.**—C. A., aged 34. A labourer. For two years he had suffered from epigastric pain, vomiting and emaciation, which had not been relieved by a gastro-enterostomy. On August 26th, 1910, the seventh to the tenth posterior roots were resected on both sides. This operation cured the pain and vomiting, but his general condition was one of such extreme debility and emaciation that he succumbed suddenly on September 21st after an attack of persistent diarrhœa. The autopsy showed that the operation wound was soundly healed.

There can be no doubt that for severe cases of gastric crises this operation affords the most radical cure that has yet been devised; but on the one hand it should be reserved for the most severe cases after other treatment has failed, and on the other it should be applied before the patient has reached the last stages of emaciation and debility.

**Cases of Spastic Paralysis.**—It is chiefly owing to the initiative of Professor Foerster that this operation has been applied to the relief of muscular spasm. Spiller and Frazier in America appear to have independently carried out the same idea. The observation which led to this practical result was that the spasm associated with lateral sclerosis disappeared when the posterior columns became involved. From this it was argued that if the cerebral control is cut off from the motor nerves the spinal reflexes produce abnormally strong muscular contractions. A considerable number of cases of spastic paralysis—both paraplegia and Little's disease—have been treated during the last two years by this operation with exceedingly gratifying results. In all cases the spasm has been immediately diminished or abolished, though permanent contractures and paralysis are not directly affected. The following original cases serve to illustrate this group.

**Case 4.**—L. R., girl, aged 16 on admission. She has had spastic hemiplegia of the type of Little's disease since early infancy. Well nourished and very intelligent. The right arm is held in extreme pronation, and the hand and digits are flexed to such a degree that the tips of the fingers touch the front of the forearm. The least attempt at correction of this deformity results in strong and painful contractions. There is spastic talipes equinus in the right foot. In May, 1910, the median nerve was forcibly stretched, and a tenotomy done for the right tendo achillis. The leg has been permanently benefited, but the hand, after a very short interval, became as contracted as ever. On December 10th, 1910, the posterior roots of the sixth, seventh, eighth cervical and first dorsal nerves were divided on the right side. The next day, and indeed directly after the operation, the hand was absolutely flaccid, within about ten days the muscles regained some of their tone, but both pain and spasm have never returned. The long-continued contraction had produced so much permanent shortening of the flexor muscles that it was necessary to resect a portion of both bones of the forearm in order to get the hand into its normal attitude. The fingers can now be fully extended by passive movements, but the loss of voluntary movement is as great as ever.

**Case 5.**—F. P., woman, aged 29. In appearance this was a very similar condition to that described in the last case. She had had a spastic contraction of the left hand and leg since infancy. There was very little actual paralysis, but in the case of the leg any dorsi-flexion of the foot caused a series of painful clonic contractions of the calf muscles, which were so violent as to cause her to fall if walking, and made her cry out in pain. On April 10th, 1911, I divided the alternate roots of all the lumbar and sacral nerves on the left side. She made a good recovery from the operation, but the condition was not improved by it. Strong dorsi-flexion of the foot still causes violent clonus, succeeded by an attack of hysterical weeping. She can walk without being thrown down, but at present there is so much ataxia in the limb that she is not conscious of any benefit. It is possible that this want of success is due to too limited division of the afferent nerve fibres, but I am inclined to attribute it to the fact that the condition is due in a large degree to a functional or psychical hysterical state.

**Case 6.**—J. C., man, aged 48, with spastic paraplegia of two years' standing. The most notable point about this man's condition was the great pain which was associated with the extreme spasm. The hips and knees were flexed, and the two legs were adducted so strongly that he had to wear pads between the knees and ankles to prevent pressure sores. The same operation was done as in the last case, but here, fortunately, success has been complete. Pain and spasm have been abolished, and after some tenotomies of the adductors and hamstring muscles had been performed, the legs could be put into fully extended and abducted position.

This very short account of my own cases will, I think, serve to show that the operation of resection of the posterior spinal nerve roots is one which (1) is devoid of much risk even when performed on patients who are very weak and ill; (2) its power of permanently relieving pain is very uncertain, because there is so large an element of psychical causation in chronic pain which cannot be eliminated; (3) it affords the most potent means of stopping the visceral crises of tabes, and will probably prove of great value in this condition if applied to suitably selected cases; (4) it cures muscular spasm associated with disease of the pyramidal tract. The benefit which such cases are likely to derive from the operation will depend upon the degree of paralysis and the absence of hysteria.

In conclusion, I would refer any who are interested in the subject to the report of the meeting of the Surgical Section of the Royal Society of Medicine (June 13th), in which I have given references to all the most important papers dealing with it.

---

